# Comparison of commercial targeted amplicon sequencing assays for human remains identification casework

**DOI:** 10.1007/s00414-024-03335-8

**Published:** 2024-10-15

**Authors:** Dennis McNevin, Jessica Watson, Kelly Grisedale, Ayusha Dahal, Corey Goodwin, Jodie Ward

**Affiliations:** 1 National DNA Program for Unidentified and Missing Persons, Australian Federal Police, Majura, ACT Australia; 2 Centre for Forensic Science, School of Mathematical & Physical Sciences, Faculty of Science, University of Technology Sydney, Ultimo, NSW Australia; 3 Thermo Fisher Scientific, Scoresby, VIC Australia

**Keywords:** Targeted amplicon sequencing, SNP genotyping, Massively parallel sequencing, Kintelligence, Ion AmpliSeq, Unidentified human remains

## Abstract

Targeted amplicon sequencing (TAS) facilitates the genotyping of forensically informative single nucleotide polymorphisms (SNPs) using massively parallel sequencing (MPS). For human remains identification, where any extracted DNA is likely to be degraded, TAS may succeed when short tandem repeat (STR) profiling using capillary electrophoresis fails. Further, as well as yielding identity information, SNPs can provide information about ancestry, phenotype, kinship and paternal lineage (Y chromosome haplotypes). Two TAS platforms were compared in this study: Ion AmpliSeq™ panels coupled with Ion Torrent sequencing on an Ion GeneStudio™ S5 Plus System, manufactured by Thermo Fisher Scientific, and the ForenSeq® Kintelligence Kit coupled with Illumina sequencing on the MiSeq FGx® Sequencing System, manufactured by QIAGEN. Four Ion AmpliSeq™ panels (Precision ID Identity, Precision ID Ancestry, DNA Phenotyping and HID Y-SNP) share 177 SNPs with the ForenSeq® Kintelligence Kit and all five were used to profile the DNA extracted from the petrous part of the temporal bone from six skeletonised cadavers. Of the 6 × 177 = 1,062 SNP genotype comparisons, 1,055 (99%) were concordant between the Ion AmpliSeq™ panels and Kintelligence Kit. Of the seven (< 1%) non-concordant SNPs, only three of them (0.3%) would have resulted in erroneous genotypes being reported as a result of allele dropout by either assay, using our optimised relative variant frequency windows for allele calling. We conclude that both the Ion AmpliSeq™ panels and the ForenSeq® Kintelligence Kit were suitable for TAS applied to the human remains in this study.

## Introduction

Human remains identification as a part of missing person investigations often involves DNA profiling from skeletal elements with long post mortem intervals (PMIs) [1]. The DNA is often of low quantity and quality [2]. As a result, short tandem repeat (STR) profiling is often not possible or results in partial profiles. Targeted amplicon sequencing (TAS) of single nucleotide polymorphisms (SNPs) is a viable alternative [3]. SNPs can be selected for incorporation into panels depending on whether they are identity informative (II SNPs), phenotype informative (PI SNPs), ancestry informative (AI SNPs), kinship informative (KI SNPs) or paternal lineage informative (Y SNPs). These panels consist of polymerase chain reaction (PCR) primer multiplexes for massively parallel sequencing (MPS) library preparation. They include the Kidd lab 92 II SNPs [4] and 55 AI SNPs [5]; the Seldin lab 128 AI SNPs [6]; the IrisPlex [7], HIrisPlex [8] and HIrisPlex-S [9] PI SNPs; and the Kayser lab high resolution Y SNPs [10], amongst others.

There are a number of competing commercial platforms for TAS of SNPs, with two of the major ones being Ion AmpliSeq™ NGS panels [11] coupled with Ion Torrent sequencing [12] (Thermo Fisher Scientific: TFS) and the ForenSeq® suite of assays coupled with Illumina sequencing-by-synthesis [13] (QIAGEN). The strategies employed by these two platforms differ in that TFS offers individual panels for II SNPs, PI SNPs, AI SNPs and Y SNPs while QIAGEN combine these in various panels. The largest of these, the ForenSeq® Kintelligence Kit, includes all of these SNP classes together with KI SNPs and X chromosome SNPs (X SNPs). SNP numbers in the Ion AmpliSeq™ panels are compared with those in the Kintelligence Kit in Table 1. The vast majority (97.8%) of amplicons included in the Kintelligence Kit are less than 150 bp [14]. Average amplicon sizes for Ion AmpliSeq™ panels are 138 bp and 127 bp for the Precision ID Identity and Precision ID Ancestry panels, respectively [15]. From this limited information, it is difficult to know what effect, if any, amplicon sizes may have on the performances of the various assays. SNP panels have much narrower size distributions than STR panels and so the effect of amplicon size on genotyping performance will be less for individual SNPs than for individual STRs. There are other technical differences between the two platforms described in Table 2, which may also have an influence on genotyping performance.

**Table 1:** Numbers of SNPs of various classes in the ForenSeq® Kintelligence Kit (QIAGEN) and Ion AmpliSeq™ (TFS) TAS panels

Marker Type	Kintelligence	Ion AmpliSeq™			
		Precision ID Identity	Precision ID Ancestry	DNA Phenotyping	HID Y-SNP
X SNPs	106	0	0	0	0
Y SNPs	85	34	0	0	780
II SNPs	94	90	0	0	0
AI SNPs	56*	0	165**	0	0
PI SNPs	24*	0	0	41**	0
KI SNPs	9,867	0	0	0	0
Totals	**10,230**	124	165	41	780
		**1,106**			

*2 SNPs overlap the Kintelligence Kit AI and PI SNPs

**4 SNPs overlap the Precision ID Ancestry Panel and Ion AmpliSeq™ DNA Phenotyping Panel

**Table 2:** Technical differences between the Kintelligence Kit coupled with Illumina sequencing-by-synthesis (QIAGEN) and Ion AmpliSeq™ panels coupled with Ion Torrent sequencing (TFS)

Step	Kintelligence Kit	Ion AmpliSeq™ panels
Library preparation	First PCR amplifies and tags the regions of interest, second PCR enriches the amplified DNA and adds adapter sequences at either end of the amplicons [16]	Partial digestion of PCR primers after PCR followed by ligation of adapter sequences at either end of the amplicons [17]
Libraries per pool	Three [16]	Eight [17] per panel, but two panels can be combined in a single pool
Surface for immobilisation of single stranded DNA	Standard Flow Cell [18]	Ion Sphere™ Particles [17]
Clonal amplification	Bridge PCR [18]	Emulsion PCR [19]
Detection of dNTP* incorporation during sequencing	Fluorescently labelled dNTP* reversible chain terminators	Electrical signal proportional to pH change on a semiconductor chip as a result of H^+^ loss after incorporation of a dNTP*
Sequencing duration	30 h [20]	17 h [20]

* deoxynucleoside triphosphate

In this study, we compared the SNP coverage (read depth), relative variant frequencies and genotype concordance of these two platforms on DNA extracted from the petrous part of the temporal bone sampled from the craniums of six cadavers with various PMIs (2 to 3 years) and decomposition environments (surface and sub-surface). The results will guide the practice of variant calling for TAS applied to human remains as well as disaster victim identification (DVI) efforts.

## Methods

The petrous part of the temporal bone was sampled from six cadavers donated to the Australian Facility for Taphonomic Experimental Research (AFTER) (Table 3) in compliance with relevant laws and institutional guidelines and approval was granted by the University of Technology Sydney (UTS) Human Research Ethics Committee (ETH18-2999/ETH21-6218). Informed consent was obtained from the six donors. A mass of 0.5 g of each petrous bone sample was pulverised and extracted using the MinElute® PCR Purification Kit (QIAGEN) [21] following total demineralisation lysis [22] and Amicon® 30 K Ultra Centrifugal Filter (Millipore) concentration [23] steps.

**Table 3:** Details of the six cadavers and the recovered petrous bone DNA used in this study

Donor	Sex	Age at death (years)	PMI (years)	Decomposition environment	DNA concentration* (ng/µL)	Degradation index (DI)**
20–01	female	33	2.6	surface	0.28	2.0
20–02	female	94	2.8	sub-surface	0.13	5.4
20–04	female	89	2.6	surface	0.13	2.3
20–06	male	67	2.5	surface	0.06	4.9
20–07	male	72	2.6	surface	0.08	1.8
20–08	male	35	2.5	sub-surface	0.55	1.8

*Quantifiler Trio™ large autosomal (LA) target

**Ratio of the DNA concentrations derived from the small autosomal target (SA) and the LA target

DNA extracted from each sample plus the extraction negative control were quantified using the Quantifiler™ Trio DNA Quantification Kit (TFS) on a QuantStudio™ 5 Real-Time PCR System (TFS) according to the manufacturer’s recommended protocol. The DNA was then diluted appropriately, according to the concentration of the large autosomal (LA) target (most sensitive to degradation), such that 0.5 ng of DNA was used as template for the GlobalFiler™ PCR Amplification Kit (except for 20–06 and 20–07 where it was only possible to supply 0.18 ng and 0.12 ng, respectively, in the 3 µL volume). The DNA from each sample was amplified for 30 cycles in a Veriti™ 96-Well Fast Thermal Cycler (TFS) and then electrophoresed on a 3500 Genetic Analyzer (TFS) with an analytical threshold of 60 relative fluorescence units (RFU) and a homozygote peak threshold of 1000 RFU, all according to the manufacturer’s recommended protocol.

Barcoded sequencing libraries were prepared for the following assays according to the manufacturers’ recommended protocols:

- ForenSeq® mtDNA Whole Genome Kit (QIAGEN) [24].
- ForenSeq® Kintelligence Kit (QIAGEN) [25] with modifications according to Watson et al*.* [26].
- Precision ID Identity Panel (TFS) [27].
- Precision ID Ancestry Panel (TFS) [28].
- Ion AmpliSeq™ DNA Phenotyping Panel [29, 30].
- Ion AmpliSeq™ HID Y-SNP Research Panel v1 [29, 31].

Extracted DNA was diluted appropriately according to the concentration of the LA target to ensure a template input amount of 1 ng for each library (0.1 ng for mtDNA). For those DNA extracts for which this was not possible (i.e. for 20–06 into the Ion AmpliSeq™ libraries and for negative controls), the maximum volume of DNA extract was added to the initial target enrichment PCR (15 µL for the Ion AmpliSeq™ libraries). Positive sequencing controls consisted of HL-60 promyeoloblast cell line (QIAGEN) for the mtDNA Whole Genome Kit, NA24385 lymphoblastoid cell line (QIAGEN) for the Kintelligence Kit [32] and AmpFℓSTR™ DNA Control 007 (TFS) for the Ion AmpliSeq™ panels while negative sequencing controls consisted of nuclease free water.

The mtDNA Whole Genome Kit libraries were prepared manually then normalised, pooled and sequenced in batches of 16 samples per standard flow cell (SFC) on a MiSeq FGx® Sequencing System (QIAGEN) according to the manufacturer’s recommended protocol unless otherwise stated. Libraries were normalised for sequencing using the Bead-Based Method (Option 1) in the manufacturer’s recommended protocol. Petrous bone samples and the extraction negative control were sequenced on the same SFC together with other samples to complete the batch. Sequencing was performed using the MiSeq FGx® Reagent Kit (QIAGEN) according to the manufacturer’s recommended protocol. Sequencing run quality metrics were assessed on the mtDNA Whole Genome Module of the Universal Analysis Software (UAS) (QIAGEN). For each sample, reads were aligned to the Revised Cambridge Reference Sequence (rCRS) and analysed using a minimum read count of 100 reads, an analytical threshold (AT) of 6% and an interpretation threshold (IT) of 50%.

The Kintelligence Kit libraries were prepared manually and then normalised, pooled and sequenced in batches of three samples per SFC on a MiSeq FGx® Sequencing System according to the manufacturer’s recommended protocol unless otherwise stated. The QuantiFluor® ONE dsDNA System (Promega) with the Quantus™ Fluorometer (Promega) was used to normalise the libraries to 0.75 ng/μL for pooling. One SFC contained the positive and negative controls while the petrous bone samples were distributed across this and other flow cells, together with other samples, consistent with emerging practice [26, 33]. Sequencing was performed using the MiSeq FGx® Reagent Kit according to the manufacturer’s recommended protocol. Different Unique Dual Index (UDI) adapters were used on successive runs to limit the effect of sample carryover. Sequencing run quality metrics were assessed on the Kintelligence Module of the UAS. Alleles were called using a modification of the default UAS analysis method with the analytical (AT) and interpretation (IT) thresholds set at 1% and the intralocus balance threshold set at 50%. Variant call files (VCFs) were generated relative to the hg19 reference genotype. A further filtering involved relative variant frequency windows for homozygotes (0.95 – 1.00) and heterozygotes (0.10 – 0.90). Variants with relative frequencies less than 0.05 were deemed sequencing error and ignored. Variants with relative frequencies in the ranges (0.05 – 0.10) and (0.90 – 0.95) were tagged as “ambiguous” [26].

The Ion AmpliSeq™ libraries were prepared on an Ion Chef™ Instrument (TFS: one Precision ID DL8 Kit per panel) and subject to 24 initial PCR cycles, except for the Y SNP panel (22 cycles). The Y SNP library was subject to a further seven PCR cycles and purified with AMPure XP Beads (Beckman Coulter) according to the manufacturer’s recommended protocol for rescuing low-yield libraries. This resulted in four pooled libraries, one per panel, with each library consisting of a barcoded positive control, negative control and six petrous bone samples. Each pooled library was quantified using the Ion Library TaqMan® Quantitation Kit (TFS) and diluted to 30 pM, which was templated and loaded onto an Ion 530™ Chip (one per library) on the Ion Chef™ Instrument according to the manufacturer’s recommended protocol. Different sets of IonCode™ Barcode Adapters were used on successive runs to limit the effect of sample carryover. Each Chip was sequenced on an Ion GeneStudio™ S5 Plus System (TFS) according to the manufacturer’s recommended protocol. Run quality metrics were assessed on the Torrent Suite Server. Alleles were called using the default variantCaller analysis method/plugin and VCFs were generated relative to the hg19 reference genotype. A further filtering involved relative variant frequency windows for homozygotes (0.9 – 1.0) and heterozygotes (0.3 – 0.7). Variants with relative frequencies less than 0.1 were deemed sequencing error and ignored. Variants with relative frequencies in the ranges (0.1 – 0.3) and (0.7 – 0.9) were tagged as “ambiguous” [34].

Final genotypes were compared for those 177 SNPs shared by both the Kintelligence Kit and the Ion AmpliSeq™ panels (Table 4). Non-concordant SNPs were identified where non-matches could not be explained by sequencing on opposite DNA strands.

**Table 4:** Numbers of SNPs of various classes shared by the ForenSeq® Kintelligence Kit (QIAGEN) and the Ion AmpliSeq™ panels (TFS)

Marker Type	Kintelligence	Ion AmpliSeq™			
		Precision ID Identity	Precision ID Ancestry	DNA Phenotyping	HID Y-SNP
X SNPs	0	0	0	0	0
Y SNPs	10	1	0	0	9
II SNPs	83	83	0	0	0
AI SNPs	56*	0	56**	0	0
PI SNPs	24*	0	0	24**	0
KI SNPs	4	4	0	0	0
Totals	**177**	88	56	24	9
		**177**			

*2 SNPs overlap the Kintelligence Kit AI and PI SNPs

**2 SNPs overlap the Precision ID Ancestry Panel and Ion AmpliSeq™ DNA Phenotyping Panel

## Results

Quantitative PCR (qPCR) positive controls were satisfactory while there was no evidence of DNA contamination in the qPCR negative control or extraction controls. DNA concentrations in the petrous bone extracts were calculated using the LA target and ranged from 0.06 ng/µL to 0.55 ng/µL (Table 3). The number of cycles required to achieve the fluorescence threshold (*C*_T_) for the internal PCR controls (IPCs) of the DNA samples in the Quantifiler™ Trio qPCR were all < 27.8 and no more than in the standards, suggesting that any PCR inhibitors not removed by the DNA extraction process were at a sufficiently low enough concentration so as not to inhibit PCR. The degradation indices (DIs) were in the range 1.8 – 5.4, suggesting that the DNA was slightly to moderately degraded (Table 3).

**Fig. 1:**
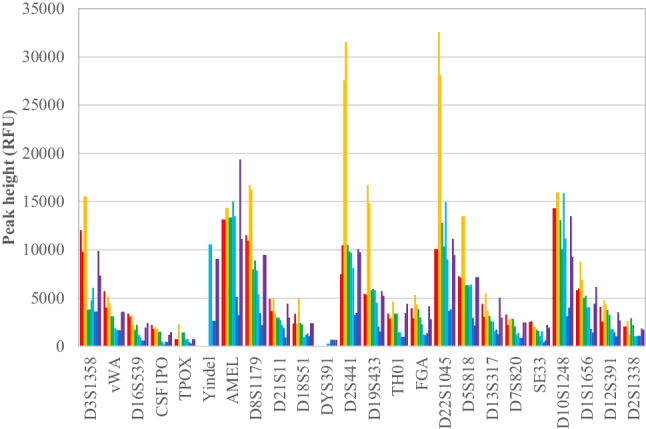
GlobalFiler™ allele peak heights for  and

Full GlobalFiler™ profiles were obtained for all six samples. Allele peak heights for each locus are shown in Fig. 1. Peak heights were generally highest for 20–02 and lowest for 20–07. All profiles were consistent with DNA that is slightly to moderately degraded.

**Fig. 2:**
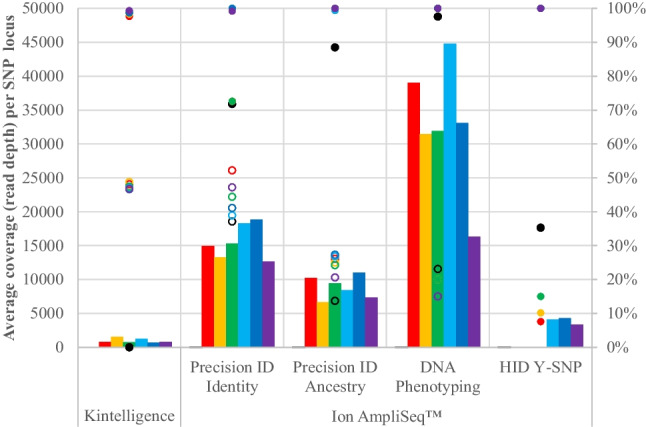
Coverage (bars, left vertical axis), percentage of SNPs typed (closed circles, right vertical axis) and observed heterozygosity (open circles, right vertical axis) for negative controls (black),  and

Coverage, percentage of SNPs typed and observed heterozygosity (proportion of heterozygous genotypes observed over all loci) for each of the six samples and for each of the Kintelligence Kit and the four Ion AmpliSeq™ panels is shown in Fig. 2. The average coverage per locus was highest for the Ion AmpliSeq™ DNA Phenotyping Panel with all 41 PI SNPs (100%) typed in all six samples. Similarly, for the Precision ID Identity Panel, all 90 II SNPs were typed in all six samples and all 34 Y SNPs were typed in the three male samples. For the Precision ID Ancestry Panel, 99.4% – 100% of the 165 AI SNPs were typed. Average coverage per locus was lowest for the Kintelligence Kit (10,230 SNPs) but, regardless, 97.6% – 99.3% of SNPs of all classes were typed. The second lowest coverage was observed for the Ion AmpliSeq™ HID Y-SNP panel with only the male cadavers (20–06, 20–07 and 20–08) having average coverage greater than 3 × . Once again, however, the lower coverage had little influence on variant call rate with all 780 Y SNPs (100%) typed in the three male samples.

Observed heterozygosity was higher for the Kintelligence Kit and Precision ID Identity Panel (37% – 52%) and lower for the Precision ID Ancestry and DNA Phenotyping Panels (15%—27%). This is in keeping with the fact that genetic markers for identity and kinship are generally selected to have high heterozygosity while genetic markers for ancestry and phenotype generally have low heterozygosity.

Positive control genotypes for the Kintelligence Kit (NA24385) and Ion AmpliSeq™ (007) panels were as expected. Only two of the 10,230 SNPs (0.02%) in the Kintelligence Kit negative control sample were detected. For the Ion AmpliSeq™ panels, however, 35%, 72%, 89% and 98% of SNPs were typed in the negative controls for the HID-Y SNP, Precision ID Identity, Precision ID Ancestry and DNA Phenotyping panels, respectively, but at much lower coverage than in samples. (Fig. 2).

**Fig. 3:**
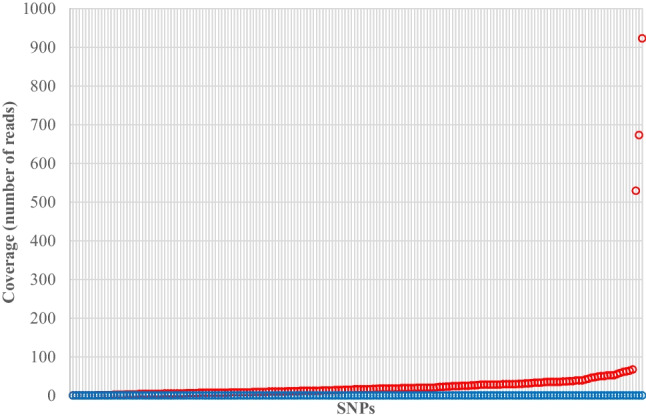
Coverage in the negative controls for the Kintelligence Kit  and Ion AmpliSeq™ panels  for SNPs shared between them. SNPs are ordered by coverage in the Ion AmpliSeq™ panels. Each vertical line represents a SNP

Coverage in the negative controls is shown in Fig. 3 for the 177 SNPs shared between the Kintelligence Kit and Ion AmpliSeq™ panels. Coverage was less than 100 × except for three SNPs with homozygous genotypes (Table 5).

**Table 5:** SNPs with high coverage (> 100 ×) in the negative controls for the Ion AmpliSeq™ panels

SNP	Panel(s)	Sub-panel(s)	Coverage	Genotype
rs1800414	Precision ID Ancestry	Kidd lab [5]	529	TT
	Ion AmpliSeq™ DNA Phenotyping	HIrisPlex-S [9]		
rs7326934	Precision ID Ancestry	Kidd lab [5]	673	GG
rs1426654	Precision ID Ancestry	Kidd lab [5]	923	GG

**Fig. 4:**
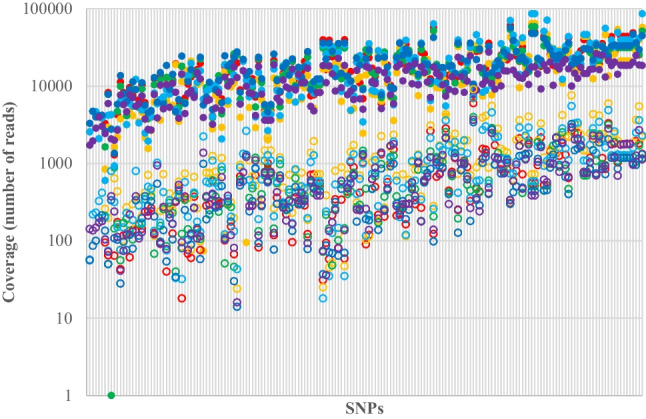
SNP coverage in the petrous bone samples for the Kintelligence Kit (open circles) and Ion AmpliSeq™ panels (closed circles). SNPs are ordered by average coverage. Each vertical line represents a SNP. Colour codes for samples are  and

**Fig. 5:**
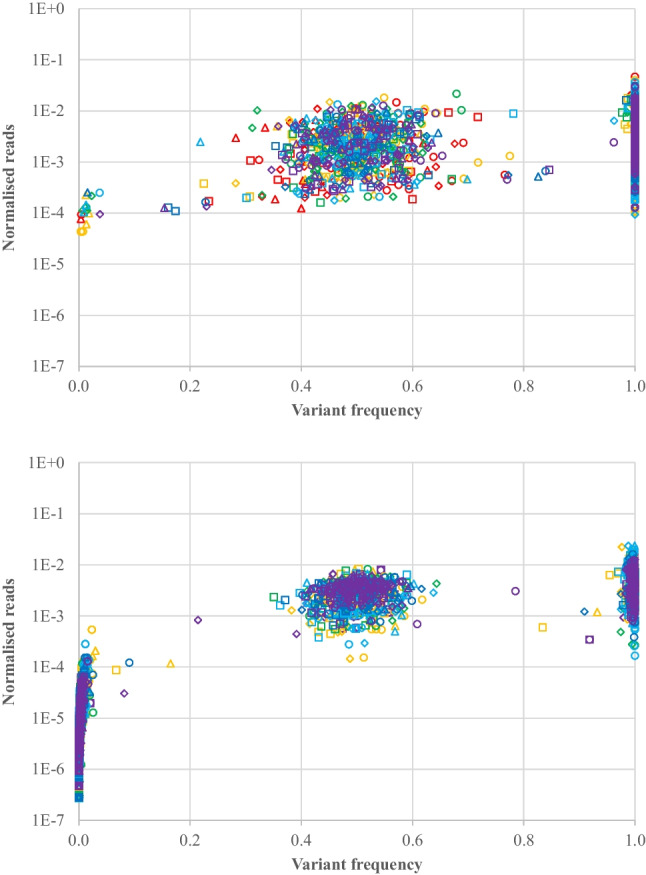
Relative variant frequency distributions in petrous bone samples for the Kintelligence Kit (top) and Ion AmpliSeq™ panels (bottom). Colour codes for samples are  and  . Allele codes are A (○), C (□), G (◊) and T (∆)

For the petrous bone samples, there were six samples with 177 shared SNPs per sample, for a total of 1,062 SNP genotypes available for comparison. Coverage in the petrous bone samples for both the Kintelligence Kit and the Ion AmpliSeq™ panels is shown in Fig. 4 where each data point represents total coverage over all alleles at one of the 1,062 genotypes for each of the Kintelligence Kit and Ion AmpliSeq™ panels. Relative variant frequency distributions for each sample are shown in Fig. 5 where each data point represents an allele from one of the 1,062 SNP genotypes for each of the Kintelligence Kit and Ion AmpliSeq™ panels. The coverage for each of the alleles in Fig. 5 has been normalised (as a proportion of total coverage over all SNPs) in order to better compare between the Kintelligence Kit and Ion AmpliSeq™ panels.

**Table 6:**
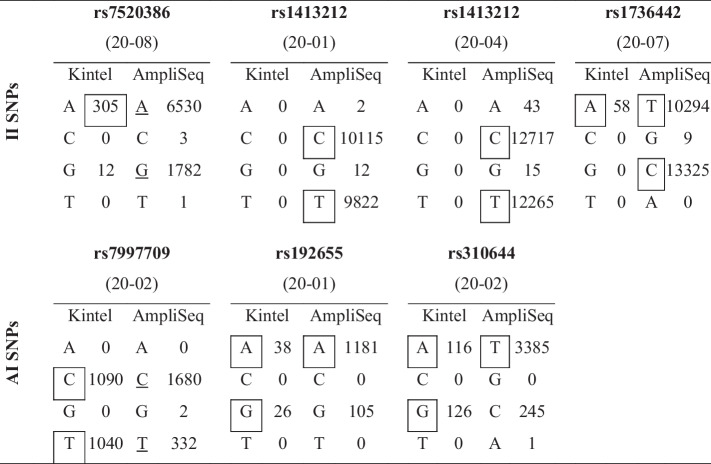
Non-concordant SNP genotypes with their called allele designations (in squares) and coverage (numbers of reads). Ambiguous variants not called by our pipeline are underlined

Of the 1,062 total SNP genotypes, 1,055 (99%) were concordant. Only seven SNPs (six of them unique) were not concordant (< 1%) and are shown in Table 6.

To investigate whether AI SNP non-concordance between the Precision ID Ancestry Panel and the Kintelligence Kit had any effect on ancestry inference, the ancestry estimates from both Converge 2.3 (TFS) with default analysis settings and the UAS were compared (Table 7). The mitochondrial DNA (mtDNA) haplogroups determined from the whole mtDNA genome sequences and the Y chromosome haplogroups determined from the 34 Y SNPs in the Precision ID Identity Panel and the 780 Y SNPs in the HID Y-SNP panel are shown in Table 8.

**Table 7:** Ancestry estimates for the six cadavers according to Converge (Precision ID Ancestry Panel) and the UAS (Kintelligence Kit)

Donor	Self-declared ancestry for self (S), mother (M) and father (F)	Converge		UAS
		Populations	Proportions (%)	Populations
20–01	Unknown	Europe Southwest Asia Oceania South Asia	91 4 4 1	Europe
20–02	Unknown	Europe South Asia Southwest Asia	97 2 1	Europe
20–04	Unknown	Europe Southwest Asia South Asia Oceania	73 26 1 1	Europe
20–06	Anglo/Celtic (S) Anglo/Celtic, NW Europe (M) NW Europe (F)	Europe Southwest Asia Africa	90 7 4	Europe
20–07	Anglo/Celtic (S) England (M) Ireland (F)	Europe Africa East Asia South Asia Southwest Asia	92 4 1 1 1	Europe
20–08	Anglo/Celtic (S) Anglo/Celtic (M) England (F)	Europe Southwest Asia	97 3	Europe

**Table 8:** Mitochondrial DNA haplogroups for the six cadavers and Y chromosome haplogroups for the three male cadavers

Donor	Sex	mtDNA haplogroup (and prevalence) [35, 36]	Y haplogroup (and prevalence) [37, 38]
20–01	female	J1b1a1 (Europe, Nth America, Sth West Asia)	N/A
20–02	female	T2a1a3a (Nth America, Sth Europe)	N/A
20–04	female	H1b2 (East Europe)	N/A
20–06	male	H1be (Europe, Nth America)	R1b (Europe)
20–07	male	T2b24a (Europe, Nth America)	R1b (Europe)
20–08	male	U4b1b1 (Nth America)	R1b (Europe)

## Discussion

DNA extracted from the six petrous bone samples was slightly to moderately degraded with no evidence of PCR inhibition. Samples 20–02 and 20–06 had the highest DIs (Table 3) and this was reflected in the most obvious “ski slope” declines in STR allele peak heights with fragment length for these samples (Fig. 1). However, this did not result in lowest coverages for the Kintelligence Kit or the Ion AmpliSeq™ panels. Sample 20–08 consistently returned lowest coverages but this sample was the least degraded (with the lowest DI). Both 20–02 and 20–08 were from buried (sub-surface) remains (Table 3) and so there was no correlation of DI with decomposition environment.

DNA was quantified for library preparation according to the LA target of the Quantifiler™ Trio Kit. The LA target (214 bp) is more prone to degradation than the SA target (80 bp) and this may explain why there was no correlation of DI with coverage. Most amplicons from the Kintelligence Kit and Ion AmpliSeq™ panels are longer than 80 bp and so a quantification based on the SA target may overestimate the amount of DNA available for amplification. Comparison of sequencing results after quantification by the SA and LA targets would be an obvious subject for further study but we have provided some evidence, at least, that quantification according to the LA target is a good strategy for at least moderately degraded human remains.

A limitation of this study is that the DNA from petrous bones was not sufficiently degraded, inhibited or of such a low concentration that STR profiling was compromised. It is possible that the Kintelligence Kit and/or the Ion AmpliSeq™ panels may perform differently in such circumstances, as the possibility of stochastic events increases, but both assays have been shown to perform well at lower concentrations [14, 39]. Even if STR profiling is possible, however, TAS may provide valuable supplementary information to aid human identification when STR profile comparisons do not achieve this, including paternal lineage, kinship, BGA and phenotypes derived from Y SNPs, KI SNPs, AI SNPs and PI SNPs, respectively.

Based on the LA target, there was sufficient DNA to provide 1 ng template input amount to both the Kintelligence Kit and Ion AmpliSeq™ panels except for 20–06 for which 0.06 ng/µL × 15 µL = 0.9 ng could be supplied to the Ion AmpliSeq™ panels. One advantage of the Kintelligence Kit is that only 1 ng of DNA in total (supplied in 25 µL) is required for library preparation. For the Ion AmpliSeq™ assays, 1 ng (supplied in 15 µL) is required for each individual panel. This means that, based on the manufacturers’ recommended input amounts, the Kintelligence Kit requires less DNA in total and can manage lower DNA concentrations (1 ng / 25 µL = 0.04 ng/µL, as opposed to 1 ng / 15 µL = 0.07 ng/µL for the Ion AmpliSeq™ assays). The consequence of this is that coverage is lower for the Kintelligence Kit because 1 ng DNA template is distributed amongst many more SNPs (Fig. 2). While there was sufficient DNA to genotype 97.6% – 99.3% of the Kintelligence Kit SNPs in this study, lower quantity and quality DNA (from human remains with longer PMIs, for example) may limit SNP coverage and reduce the effectiveness of the Kintelligence Kit. Of course, there are other differences between the Kintelligence Kit and the Ion AmpliSeq™ panels (Table 1 and Table 2). The 9,867 KI SNPs in the Kintelligence Kit means that it has much greater reach for forensic kinship analysis. On the other hand, there are more AI SNPs in the Precision ID Ancestry Panel, more PI SNPs in the DNA Phenotyping Panel and more Y SNPs in the HID Y-SNP panel than in the Kintelligence Kit. This means that the Ion AmpliSeq™ panels can potentially provide greater resolution for inference of biogeographical ancestry (BGA) and Y haplogroups as well as offering skin colour inference in addition to eye and hair colour inference.

Other factors that may influence any decision to adopt either the Kintelligence Kit or the Ion AmpliSeq™ panels include instrument availability, hands on time in the laboratory, cost per sample and total cost. These are surveyed by Gettings et al*.* [20] in Table S7 using the “maximum batch size recommended by the manufacturer”. The Precision ID Identity Panel (as a representative Ion AmpliSeq™ panel) ranges from US$171 to US$225 per sample for post-mortem samples (lower if two panels are combined in a single pool), depending on what combination of library preparation and Ion Chip is employed. This can be compared with US$1,100 to US$2,005 for the Kintelligence HT Kit and the Kintelligence Kit, respectively (the HT kit is the high throughput version). While the Kintelligence Kit is five to ten times more expensive per sample, it includes not just II SNPs, but also AI SNPs, PI SNPs and Y SNPs that would require the three additional Ion AmpliSeq™ panels. Of course, Kintelligence also allows for more extensive kinship analysis because it includes nearly 10,000 KI SNPs. Batch processing time is longer for the Kintelligence Kit due mostly to sequencing time on the MiSeq FGx® [20].

Sequencing coverage in the controls and petrous bone samples reflected differences in the numbers of SNPs in the two platforms. The Kintelligence Kit has 80 × , 60 × , 250 × and 13 × more SNPs than the Precision ID Identity, Precision ID Ancestry, DNA Phenotyping and HID Y SNP panels, respectively (Table 1). Consistent with this, the coverage for the Kintelligence Kit was one to two orders of magnitude less than the coverage for the Ion AmpliSeq™ panels (Fig. 4). While there was very low coverage (relative to samples) for most SNPs in the negative control for the Ion AmpliSeq™ panels, all except two SNPs received no coverage at all for the Kintelligence Kit (Fig. 2). Of the 1,106 SNPs in all four of the Ion AmpliSeq™ panels, 509 (46%) received no coverage in the negative control and 1,080 (98%) received less than 100 reads which is at least an order of magnitude less than the coverage for the vast majority of SNPs in the six petrous bone samples (Fig. 4). This means that any background coverage had little bearing on the relative variant frequencies (and genotypes) for these samples. This coverage can be thought of as baseline noise in a signal-to-noise ratio which is elevated for the Ion AmpliSeq™ panels (relative to the Kintelligence Kit) because the coverage (signal) is much higher.

Of the 177 shared SNPs, three of them from the Precision ID Ancestry Panel (one shared with the DNA Phenotyping panel) received high coverage (> 100 ×) in the negative control for one allele (Table 5). It is possible that there was a contamination event for the Precision ID Ancestry library, but it is unusual that it should be restricted to three SNPs. Regardless, none of these three SNPs resulted in a non-concordant allele call between the Ion AmpliSeq™ panels and the Kintelligence Kit for any of the six petrous bone samples. This means that if these alleles contaminated all samples in the Ion AmpliSeq™ panels, they did not push variant frequencies out of the homozygote and heterozygote variant frequency windows. For this particular case, however, it would be best practice to exclude these SNPs from further analysis if they formed part of operational casework.

The performance of any TAS assay can be visualised using relative variant frequency distributions (Fig. 5). An ideal TAS assay would have only two small clusters: one in the middle at a relative variant frequency of 0.5, representing heterozygotes, and one on the right-hand side at a relative variant frequency of 1.0, representing homozygotes. The coverage for the homozygous alleles would be exactly twice that for the heterozygous alleles in this ideal assay. In reality, however, there is variation in coverage (and, therefore, variation in relative variant frequencies) such that there are dispersed clusters for heterozygotes and homozygotes. Firstly, not all SNPs receive the same total coverage because of different primer efficiencies for each SNP in the PCR multiplex. Secondly, for each SNP, not all alleles receive the same coverage because of stochastic variation, especially for low template amount DNA. We also see a cluster at the bottom left at a relative variant frequency near 0 which represents low frequency, low coverage variants. This represents sequencing error.

**Fig. 6:**
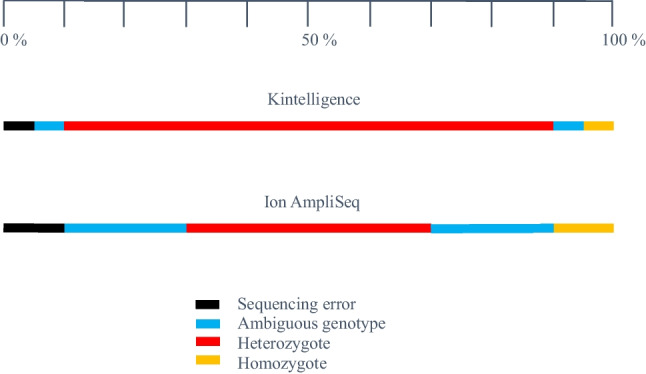
Relative variant frequency windows used in this study to define genotypes for the Kintelligence Kit and Ion AmpliSeq™ panels

The relative variant frequency distributions in Fig. 5 show the differences between the Kintelligence Kit and Ion AmpliSeq™ panels. The heterozygote cluster is tighter (smaller) for the Ion AmpliSeq™ panels, which is probably a result of the higher coverage they provide; this is, in turn, a result of the smaller numbers of SNPs in the PCR multiplexes. The heterozygote cluster for the Kintelligence Kit is more dispersed because of the much larger numbers of SNPs that require coverage. Conversely, there is more sequencing error for the Ion AmpliSeq™ panels (bottom left clusters), again, probably because of the higher coverage. What is important is that the three clusters can be differentiated such that any allele can be assigned as part of a homozygote or heterozygote genotype, or as sequencing error. This means that different relative variant frequency windows must be employed for the two platforms. For the Ion AmpliSeq™ panels, a smaller window can be used to define heterozygotes, but a window for sequencing error must also be defined. For the Kintelligence Kit, a wider window for heterozygotes can be tolerated because there is little sequencing error. The relative variant frequency windows employed in this study are shown in Fig. 6. Figure 5 shows that while most variants fall within the three clusters for homozygotes, heterozygotes and sequencing error, some fall between the clusters. These were deemed “ambiguous” genotypes, characterised by unbalanced relative variant frequencies and are not reported in our pipeline.

Examination of Table 6 shows that there were four reasons for non-concordance between the Kintelligence Kit and Ion AmpliSeq™ panels for seven SNPs (six of them unique). For two SNPs (rs7997709 in 20–02 and rs7520386 in 20–08) there were ambiguous genotypes for the Ion AmpliSeq™ panels, but not for the Kintelligence Kit. Because they were ambiguous, the two Ion AmpliSeq™ genotypes would be removed from further analysis so that no erroneous genotype would be reported. For one SNP (rs1413212) in two different samples (20–01 and 20–04), there was locus dropout for the Kintelligence Kit but not for the Precision ID Identity Panel. Again, no erroneous genotype would be reported. For two SNPs (rs192655 in 20–01 and rs310644 in 20–02), there was allele dropout for the Precision ID Ancestry Panel but not for the Kintelligence Kit. For one SNP (rs1736442 in 20–07) there was allele dropout for the Kintelligence Kit but not for the Precision ID Identity Panel. Therefore, it is only these last three SNPs for which erroneous genotypes would be reported in our pipeline.

Ancestry estimates from both Converge (Precision ID Ancestry Panel) and the UAS (Kintelligence Kit) were compared to see if the AI SNP non-concordance resulted in any differences. Because they use different algorithms and different numbers of AI SNPs, it is impossible to know definitively if any differences are due to genotype non-concordance but examination of Table 7 suggests that any differences were minimal. All six cadavers appeared in the European cluster of the UAS principal components analysis (PCA) plot. Five of the six cadavers were estimated to have over 90% of their ancestry derived from a European ancestral population by Converge with only 20–04 predicted to have 73% from a European population and 26% from a Southwest Asian population. This could be the signature of an individual with Southern European and/or Mediterranean ancestry (unfortunately, the self-declared ancestry for this cadaver was unknown).

The Y haplogroups for all three male cadavers were R1b, which is prevalent in Europe (Table 8). The mtDNA haplogroups were also predominantly European or North American (where many individuals with European ancestry reside). The ancestries inferred by both Converge and the UAS are consistent with these haplogroups as well as with the known self-declared ancestries for cadavers 20–06, 20–07 and 20–08.

## Conclusion

TAS assays with medium to large SNP multiplexes can be used to aid human remains identification and missing persons investigations when STR profiling either does not work or is not sufficient for identifying genetic relatives beyond first or second order. We investigated two SNP genotyping platforms with two different strategies: a large, all-in-one panel including all classes of SNPs (ForenSeq® Kintelligence Kit genotyped on a MiSeq FGx®) and four separate smaller panels of SNPs (Ion AmpliSeq™, all sequenced on an Ion GeneStudio S5 Plus). There were 177 SNPs in common between these two platforms. They were compared by examining SNP genotypes from DNA extracted from the petrous parts of the temporal bones recovered from six human cadavers exposed to differing environmental conditions for up to three years. Three SNPs (rs1800414, rs7326934 and rs1426654) received high coverage in the Ion AmpliSeq™ negative controls, but none of them resulted in non-concordant allele calls when comparing between Ion AmpliSeq™ panels and the ForenSeq® Kintelligence Kit in any sample. Less than 1% of SNP genotypes were not concordant between the Kintelligence Kit and Ion AmpliSeq™ panels and only three SNP comparisons (rs192655 in 20–01, rs310644 in 20–02 and rs1736442 in 20–07) of a total of 1,062 would result in reporting of erroneous genotypes because of allele dropout in either the Kintelligence Kit or Ion AmpliSeq™ panels. This minimal non-concordance did not result in any difference in ancestry inference using Converge and the UAS and both were consistent with known self-declared ancestries as well as with mtDNA and Y chromosome haplogroups. Relative variant frequency windows are important thresholds for optimisation of TAS panels and can be used to identify ambiguous genotypes that should not be reported, avoiding erroneous genotyping.

## Data Availability

The datasets generated and analysed during the current study are not publicly available because of the conditions of ethical approval but are available from the corresponding author on reasonable request.
